# Locally Applied Valproate Enhances Survival in Rats after Neocortical Treatment with Tetanus Toxin and Cobalt Chloride

**DOI:** 10.1155/2013/497485

**Published:** 2013-09-14

**Authors:** Dirk-Matthias Altenmüller, Jonas M. Hebel, Michael P. Rassner, Silvanie Volz, Thomas M. Freiman, Thomas J. Feuerstein, Josef Zentner

**Affiliations:** ^1^Epilepsy Center, Department of Neurosurgery, University Hospital Freiburg, Breisacher Straße 64, 79106 Freiburg, Germany; ^2^Section of Clinical Neuropharmacology, Department of Neurosurgery, University Hospital Freiburg, Breisacher Straße 64, 79106 Freiburg, Germany; ^3^Department of Neurosurgery, University Hospital Freiburg, Breisacher Straße 64, 79106 Freiburg, Germany

## Abstract

*Purpose*. In neocortical epilepsies not satisfactorily responsive to systemic antiepileptic drug therapy, local application of antiepileptic agents onto the epileptic focus may enhance treatment efficacy and tolerability. We describe the effects of focally applied valproate (VPA) in a newly emerging rat model of neocortical epilepsy induced by tetanus toxin (TeT) plus cobalt chloride (CoCl_2_). *Methods*. In rats, VPA (*n* = 5) or sodium chloride (NaCl) (*n* = 5) containing polycaprolactone (PCL) implants were applied onto the right motor cortex treated before with a triple injection of 75 ng TeT plus 15 mg CoCl_2_. Video-EEG monitoring was performed with intracortical depth electrodes. *Results*. All rats randomized to the NaCl group died within one week after surgery. In contrast, the rats treated with local VPA survived significantly longer (*P* < 0.01). In both groups, witnessed deaths occurred in the context of seizures. At least 3/4 of the rats surviving the first postoperative day developed neocortical epilepsy with recurrent spontaneous seizures. *Conclusions*. The novel TeT/CoCl_2_ approach targets at a new model of neocortical epilepsy in rats and allows the investigation of local epilepsy therapy strategies. In this vehicle-controlled study, local application of VPA significantly enhanced survival in rats, possibly by focal antiepileptic or antiepileptogenic mechanisms.

## 1. Introduction

The effective treatment of focal neocortical epilepsies not satisfactorily responsive to systemic antiepileptic drug (AED) therapy remains a considerable clinical challenge. Surgical removal of the epileptogenic zone is not an ideal alternative option to pharmacotherapy if eloquent brain areas are involved. Moreover, oral administration of AEDs at dosages high enough for adequate seizure control may be associated with intolerable systemic side effects. Emerging therapeutic strategies aiming at enhanced efficacy and tolerability therefore include the local application of AEDs directly onto the individually identified epileptic focus. To systematically evaluate such a local epilepsy therapy in animals, we have recently constructed particular valproate (VPA) polycaprolactone (PCL) implants and determined their VPA release kinetics [1].

The development of novel antiepileptic agents and therapeutic strategies in humans relies on the preclinical application of adequate animal models (for review, see [2]). However, the establishment of a reliable and valid animal model of focal neocortical epilepsy with recurrent spontaneous seizures is not trivial [3]. Tamargo et al. [4] first showed the proof of principle of local neocortical epilepsy therapy using phenytoin polymers in a cobalt-induced epilepsy model in rats [5–7]. Regrettably, the time course of cobalt-induced neocortical epilepsy is limited, typically leading to extinction of the cobalt focus within three weeks. Furthermore, when applying 20 mg cobalt chloride (CoCl_2_) powder to the cortex, the authors had to deal with a considerable attrition in the number of rats. In contrast to cobalt-based focal epilepsy models, Nilsen et al. [8]. reported long-term and frequently recurrent unprovoked seizures in rats after neocortical injection of 25–50 ng tetanus toxin (TeT). However, we failed to reproduce reliably spontaneous seizures in the TeT model of focal neocortical epilepsy. This was true also after increasing the dosage of TeT substantially. 

We therefore intended to develop a new rat model of focal neocortical epilepsy by combining a triple injection of TeT in the primary motor cortex together with the cortical application of CoCl_2_. Preliminary results with this novel methodological approach encouraged us to examine the antiepileptic and possibly antiepileptogenic effects of VPA applied via PCL implants at the neocortical site treated with TeT and CoCl_2_. We here report on the methods and results of a vehicle-controlled pilot study. We hypothesized that the local application of VPA onto a neocortical epileptic focus, induced by TeT and CoCl_2_, might reduce interictal epileptic activity in intracranial EEG as well as video documented seizure frequency and severity. Consequently, a reduction of seizure-related deaths could possibly be expected in rats focally treated with VPA.

## 2. Materials and Methods

All animal procedures were carried out according to the National Institute of Health Guide for the Care and Use of Laboratory Animals and were approved by the local ethics committee (Regierungspräsidium Freiburg, G-09/10).

### 2.1. Materials

TeT was provided by Quadratech Diagnostics Ltd (Epsom, UK) at a working concentration of 100 ng/*μ*L (sterile distilled water with 2% bovine serum albumin).

Cobalt(II) chloride hexahydrate (Sigma-Aldrich Chemie GmbH, Steinheim, Germany) was dissolved in water at portions of 15 mg each. Evaporation of this solution in standard Eppendorf tubes (Eppendorf Vertrieb Deutschland GmbH; Wesseling-Berzdorf, Germany) resulted in round slices of CoCl_2_ with a diameter of about 4 mm.

Electrodes consisted of teflon-coated silver wires (0.38 mm diameter; World Precision Instruments, Sarasota, USA) with deinsulated tips. 

PCL implants (diameter 4.9 mm–5.1 mm; thickness 0.9 mm–1.1 mm) containing either VPA or sodium chloride (NaCl) 10% w/w were produced by a novel 3D-bioplotting technology (Freiburg Materials Research Center, University of Freiburg, Freiburg, Germany), leading to continuous local VPA release [1].

### 2.2. Animals

Ten male Sprague-Dawley rats (University of Freiburg, Freiburg, Germany) at a mean age of 100 days and a mean weight of 250 g on the day of surgery were included. Animals were kept in standard animal facilities (one rat per cage after surgery) under controlled temperature of 21°C, with a 12-h light/dark cycle and free access to food and water.

### 2.3. Surgical Procedures

Rats were anesthetized with 5 vol% isoflurane and placed in a small animal stereotaxic frame assembly (David Kopf Instruments, Tujunga, CA). Further surgery was performed with 1.5–2.5 vol% isoflurane and 1-2 L/min oxygen. After exposing the skull, hydrogen peroxide was applied, using its haemostatic and antiseptic effect to prepare the operating field. During surgery, 5 mL NaCl 0.9% were injected subcutaneously (s.c.) for compensation of a possible fluid loss. Directly after surgery, 0.015 mg buprenorphine was given s.c. for analgesia. 

All coordinates given below for local TeT injection or electrode position relate to the bregma and were determined according to a stereotaxic atlas of the rat brain [9].

#### 2.3.1. Preparation of the Neocortical Focus

A hole (5 mm diameter) was carefully countersunk with a mill in the skull above the right primary motor cortex (anterior to the bregma, with edges 1 mm lateral to the midline and bordering the coronal suture). Care was taken not to injure the cortical surface. While drilling, the mill was constantly cooled with saline solution. The meninges were removed with a dural hook. Drying out of the cortical tissue was prevented by regular administration of saline solution. 

A total amount of 225 ng TeT in three volumes of 0.75 *μ*L each was injected stereotaxically 1 mm below the neocortical surface (1st injection: 1.1 mm anterior, −2.5 mm lateral; 2nd injection: 2.1 mm anterior, −1.5 mm lateral; 3rd injection: 2.1 mm anterior, −3.5 mm lateral), using a 2 *μ*L Hamilton syringe (Carl Roth GmbH & Co. KG, Karlsruhe, Germany). 

Afterwards, a 15 mg round slice of CoCl_2_ was laid onto the motor cortex and slightly pushed down by a PCL implant (containing either VPA or NaCl) by which the bore was sealed.

#### 2.3.2. Electrode Placement

**Figure 1:**
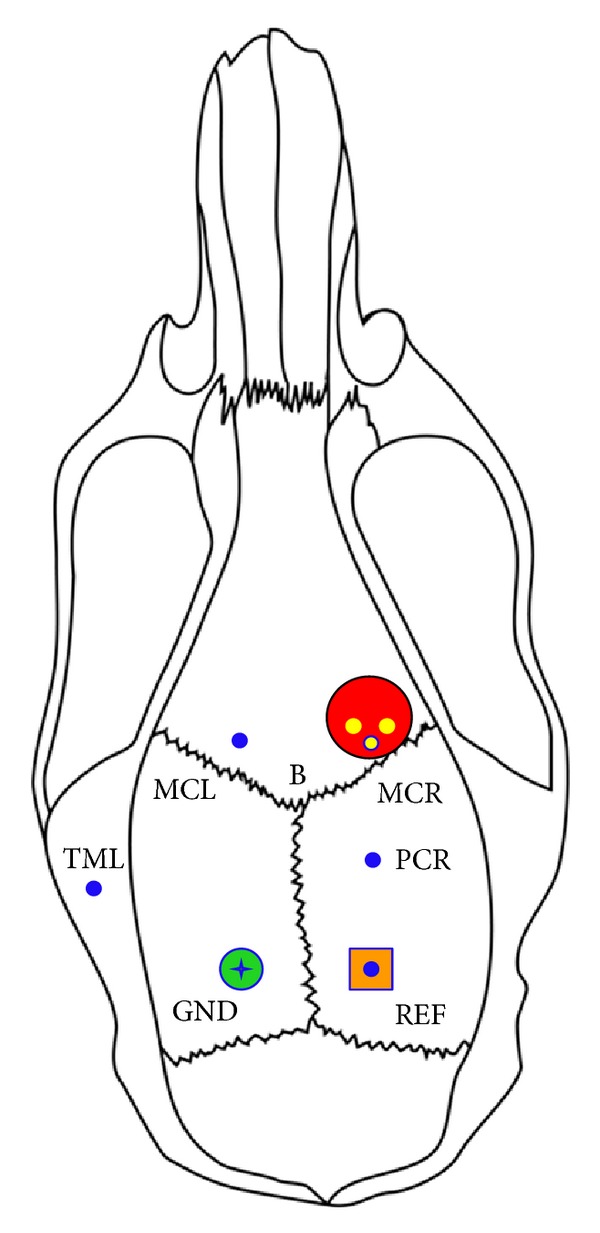
Schematic of the locations of three intracortical (MCR, right motor cortex; MCL, left motor cortex; PCR, right parietal cortex), one intramuscular (TML, left temporal muscle), one reference (REF), and one ground (GND) electrodes (blue circles) with respect to the rat skull (B = bregma), to the tetanus toxin injection sites (three small yellow circles) and to the application site (large red circle) of cobalt chloride and polycaprolactone implants loaded with valproate or sodium chloride.

For electrocorticography (ECoG) und electromyography (EMG) recordings, six electrodes were placed in each rat (Figure 1). Three intracortical depth electrodes were implanted, targeting intrafocally the right primary motor cortex (MCR; 1.1 mm anterior, −2.5 lateral) and extrafocally both the left motor cortex (MCL; 1.1 mm anterior, 2.5 mm lateral) and the right parietal cortex (PCR; −3.0 mm posterior, −2.5 mm lateral). In order to facilitate the implantation of the electrode MCR, a small hole was predrilled into the PCL implant before sealing the bore. An EMG electrode was jabbed into the left temporal muscle (TML). Primarily designed as an indicator for epileptic motor activity contralateral to the induced seizure focus, this electrode also proved to be helpful in sorting out movement artifacts. For the reference electrode (REF), a special pin was designed, which was placed epidurally within the calvarium after drilling a small and shallow hole into the skull overlying the posterior part of the right hemisphere (−7.0 mm posterior, −2.5 mm lateral). After fixation, an electrode was plugged into the pin. Contralaterally, another electrode was attached with an epidural and intraosseous stainless steel screw which served as ground (GND). 

All electrodes were linked to a 6-pin plug connector (Bürklin OHG, Oberhaching, Germany) with two anchor bars. To assure permanent fixation of the electrodes, the operating field was sealed with dental acrylate (Paladur; Heraeus Holding GmbH, Hanau, Germany).

### 2.4. Video-ECoG Recordings

For simultaneous recording of the postoperative behaviour of the rats, of the ECoG and of the EMG, rats were placed in individual transparent cages. 

The cranial electrode assembly was connected to an MPA8I preamplifier (Multi Channel Systems MCS GmbH, Reutlingen, Germany), a slip-ring (Air Précision SA, Le Plessis-Robinson, France), and a PGA32 amplifier (Multi Channel Systems MCS GmbH, Reutlingen, Germany), coupled to an analog-to-digital converter CED Power1401 (Cambridge Electronic Design Limited, Cambridge, UK). Signals were amplified (×5000), filtered (band-pass of 1 Hz–5 kHz), and digitized at a sampling rate of 5–10 kHz. Data were stored and then visually analyzed in a referential montage to REF using the CED Spike2 software, version 5 (Cambridge Electronic Design Limited, Cambridge, UK). 

The behaviour of the rats was monitored simultaneously with a digital high definition video camera recorder (model HDR-HC5E; Sony Corporation, Tokyo, Japan) and synchronized with the ECoG and EMG traces. 

From the first day after surgery, animals were scheduled for video-ECoG monitoring every three days for a period of three weeks and at least once a week thereafter. Each monitoring session lasted at least two hours. ECoG sections disrupted by relevant movement or other artifacts were excluded from the evaluation.

### 2.5. Data Analysis

ECoG and video recordings were visually analyzed by an epileptologist (blinded regarding treatment until day 21 after surgery) and an assistant trained in ECoG interpretation in rats. The analysis focused on a qualitative and quantitative evaluation of both interictal and ictal activity and seizure semiology. Efforts were made not to mistake artifacts for epileptic activity. 

Interictal epileptic activity was defined as unambiguous epileptiform potentials characterized by single or multiple spikes followed by a slow wave and clearly interrupting background activity with respect to amplitude and frequency. 

By definition, ictal seizure patterns consisted of runs of repetitive spiking or rhythmic activity with a dynamic evolution in frequency and topography. Seizure patterns without any overt clinical manifestation as assessed by simultaneous video recordings were defined as subclinical. 

After identification, epileptiform potentials and subclinical and clinical seizures were counted. Additionally, seizure duration was determined, and semiology was correlated with ictal ECoG topography.

### 2.6. Local Treatment

The PCL implants closed the bore above the right primary motor cortex and contained either VPA or NaCl 10% w/w. After regional diffusion of CoCl_2_ into the tissue, the PCL implants had direct contact to the neocortex and released their content continuously towards the tissue. The assignment of the individual rats to local VPA (*n* = 5) or NaCl (*n* = 5) was random.

### 2.7. Statistical Analysis

Rat survival as a function of treatment was determined as Kaplan-Meier estimates, and differences in the survival curves were evaluated with this method. There were no censored data. A *P* value less than 0.05 was considered significant.

## 3. Results

### 3.1. Mortality

**Figure 2:**
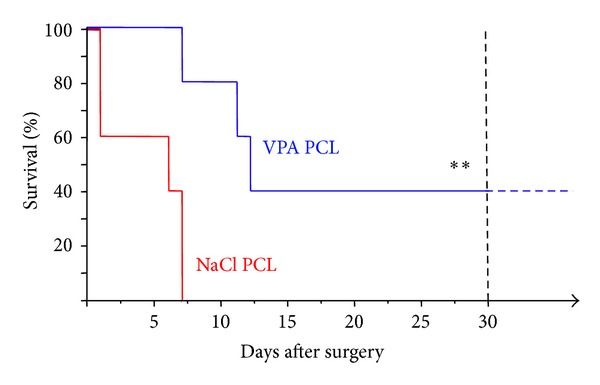
Kaplan-Meier estimates of postoperative survival stratified by local treatment with either valproate (VPA) or sodium chloride (NaCl) polycaprolactone (PCL) implants. Animals of the VPA group survived significantly longer (**, *P* < 0.01).

All TeT/CoCl_2_ rats randomized to the NaCl group died within one week after surgery (two animals on days 1 and 7, respectively; one animal on day 6). In contrast, the TeT/CoCl_2_ rats treated with VPA survived significantly longer (*P* < 0.01), with postoperative death occurring on days 7, 11, and 12 or sacrifice after more than 5 months (*n* = 2). A plot of the Kaplan-Meier estimate of the survival function is shown in Figure 2. 

When witnessed (*n* = 3), exitus took place during status epilepticus (NaCl group, day 7) or was probably seizure-related (NaCl group, day 7; VPA group, day 11). Neither in the VPA nor in the NaCl group any relevant complication had occurred along with the invariable surgical procedures. 

### 3.2. Video-ECoG Recordings

No video-ECoG data are available for the two rats of the NaCl group which died the day following surgery. 

After discarding periods with ECoG artifacts, ECoG recordings of good or sufficient quality in the remaining eight rats were sampled between days 1 and 30 after surgery. These recordings had an average total duration of 460 minutes (range: 140–1460 minutes) and were systematically analyzed. The interanimal variability of the total duration of the individually analyzed ECoG recordings is explained by differences in the extent of artifacts and different survival times. The screening of video-ECoG recordings beyond 30 postoperative days in two rats of the VPA group did not yield any subclinical or clinical seizures or a relevant further change of interictal epileptic activity. 

In the resting ECoG, a physiological theta rhythm more or less intermingled with alpha and delta activity was prevailing in all animals. 

**Figure 3:**
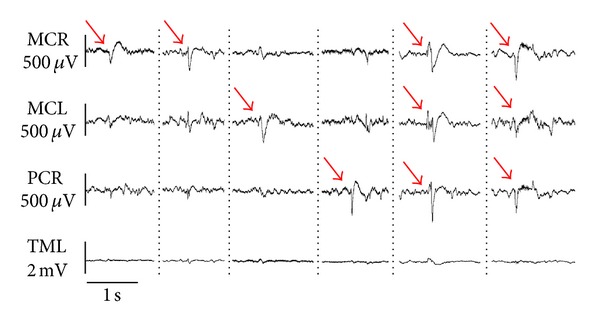
Interictal electrocorticography. Typical epileptiform potentials (red arrows) with different locations (see Figure 1). MCR, right motor cortex; MCL, left motor cortex; PCR, right parietal cortex; TML, left temporal muscle.

All TeT/CoCl_2_ rats with available ECoG recordings showed clear-cut interictal epileptiform potentials. Examples of their interindividually varying specific appearance are depicted in Figure 3. The peak-to-peak amplitude of the epileptiform potentials commonly was in the range of 150 to 600 *μ*V.

In one animal (NaCl group, day 6), the leading interictal epileptic focus was identified at the electrode within the right motor cortex treated with TeT and CoCl_2_. In all other rats, epileptiform potentials were predominantly located at electrodes outside the TeT/CoCl_2_ application site or were distributed diffusely. 

**Figure 4:**
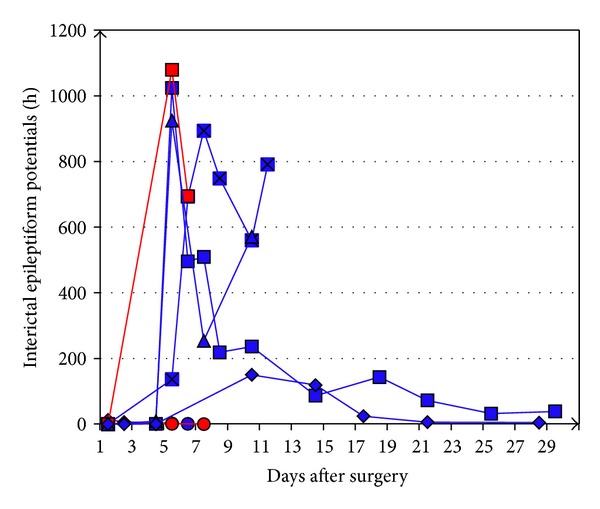
Mean frequency of interictal epileptiform potentials per hour (several recording periods per day) in individual rats (blue, rats of the valproate group; red, rats of the sodium chloride group) during the postoperative course.

Their frequency of occurrence varied interindividually but constantly reached a peak between days 5 and 7 (range: days 1–10) after surgery (Figure 4). In this period, up to 1950 epileptiform potentials per hour were counted in a single recording period of a rat of the NaCl group. 

Definite electroencephalographic seizure patterns were documented during sampling in 2 out of 3 rats of the NaCl group and in 4 out of 5 rats of the VPA group. They occurred spontaneously beyond the acute surgery and toxin administration period and were often clearly associated with clinical seizure symptoms in all affected rats. Thus, at least 75% of the TeT/CoCl_2_-treated rats surviving the first postoperative day developed epilepsy, by definition. The latency between TeT/CoCl_2_ application and epilepsy onset lay in the range between days 1 and 5 but could not be defined precisely as video-ECoG monitoring was noncontinuous. Therefore, the first unprovoked seizure in a given rat may have been missed. In the two long-term surviving rats of the VPA group, the latest seizures sampled during repeated monitoring epochs occurred on days 8 and 11, respectively. 

**Figure 5:**
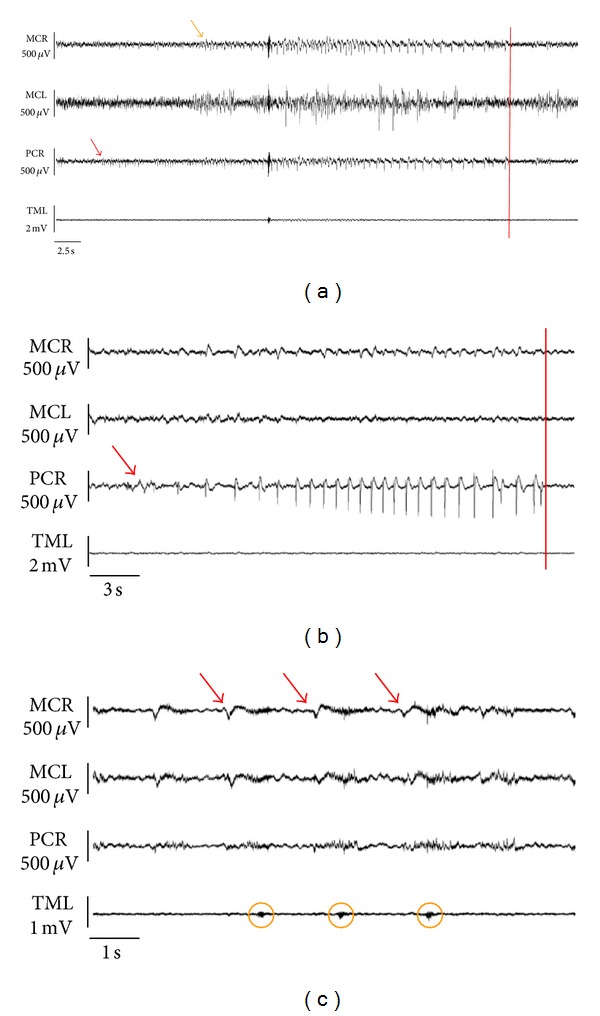
Ictal electrocorticography. (a) Subclinical seizure pattern. Focal seizure onset (red arrow) in PCR, ipsilateral propagation (orange arrow) to MCR. (b) Clinical seizure pattern associated with clonic jerks of the left hind limb. Focal seizure onset (red arrow) and end registered in PCR. Note the absence of ictal electromyographic activity in the left temporal muscle. (c) Detail of clinical seizure pattern. Note the close correlation between repetitive ictal bursts in MCR (red arrows) and contralateral facial twitching (orange circles) as evidenced by temporal electromyography (TML). MCR, right motor cortex; MCL, left motor cortex; PCR, right parietal cortex; TML, left temporal muscle; red bars, seizure end.

ECoG seizure patterns consisted of repetitive spiking or rhythmic activity (most commonly in the delta or theta range) with clear evolution in rhythm frequency and spatial distribution and with durations between 2 and 189 seconds. Their specific appearance varied among individual animals. Examples of individual subclinical and clinical ECoG seizure patterns are shown in Figure 5. 

Seizure onset, as determined by the three depth electrodes, was unequivocally localized at the TeT/CoCl_2_ application site only twice (NaCl group, day 6; VPA group, day 4). Predominantly, ECoG seizure patterns involved initially the ipsilateral electrode outside the TeT/CoCl_2_ application site or were distributed diffusely at all intracranial electrodes from the beginning onwards. 

**Figure 6:**
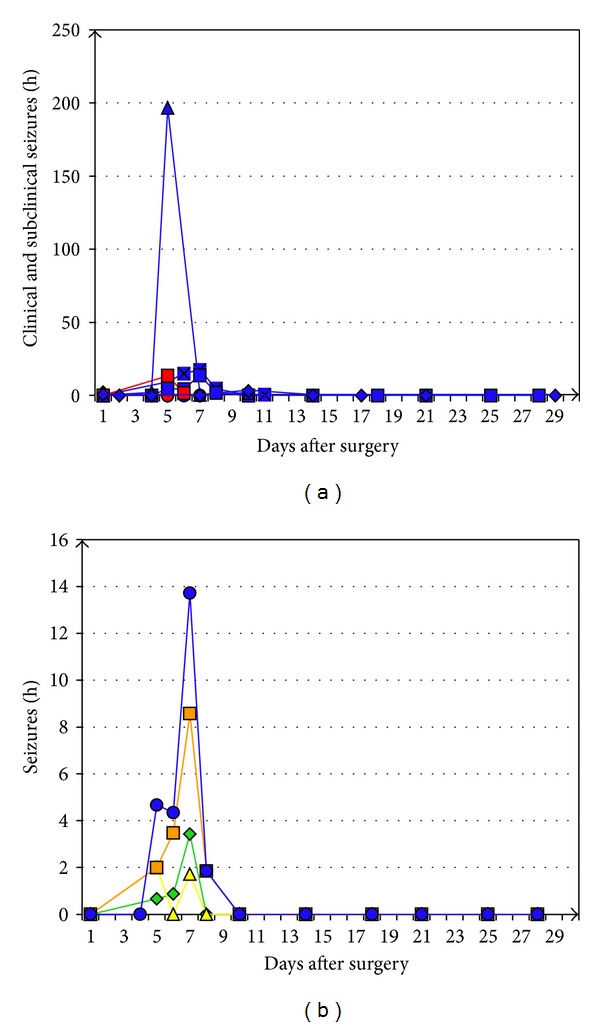
(a) Mean frequency of total subclinical and clinical seizures per hour in individual rats (blue, rats of the valproate (VPA) group; red, rats of the sodium chloride group) during the postoperative course. (b) Further differentiation of the time course of subclinical (green), not generalized focal (yellow), and secondary generalized tonic-clonic (orange) seizures in an exemplary rat of the VPA group (blue, total subclinical and clinical seizures).

The frequency of subclinical ECoG seizure patterns and patterns associated with clinically overt seizures varied interindividually. Typically, there was a peak between postoperative days 5 and 8 (range: days 1–10; Figure 6). Some rats experienced up to 10 or even more than 50 clinical seizures per hour.

In all animals, the video-analyzed seizure semiology was characterized by rhythmic clonic jerks or—more rarely—tonic posturing predominantly involving the head and the limbs contralateral to the TeT/CoCl_2_ application site. Duration of clinical seizures commonly was between 20 and 60 seconds (range: 6–189 seconds). One rat of the VPA group, additionally, exhibited intermittent ictal left-sided facial twitching (Figure 5(c)). Two rats of the VPA group experienced secondary generalized tonic-clonic seizures. 

A statistical comparison of the two treatment groups with respect to qualitative or quantitative aspects of interictal and ictal ECoG or semiology was not possible, as only few rats treated with NaCl were eligible for long-term video-ECoG. 

**Figure 7:**
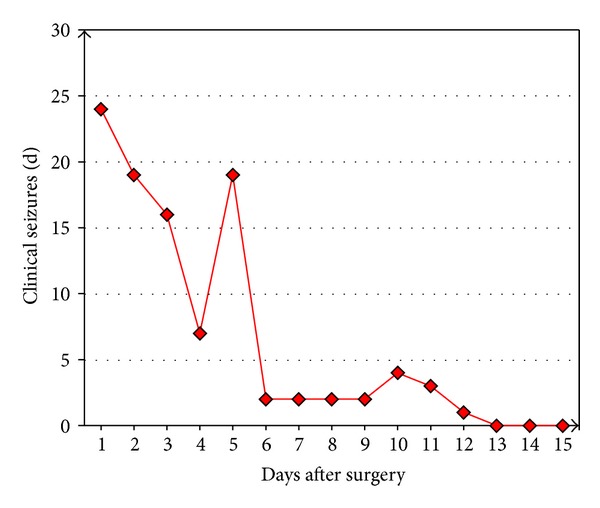
Frequency course of clinical seizures in an additional rat continuously monitored by video-ECoG after neocortical administration of 187.5 ng TeT and 7.4 mg CoCl_2_. No local valproate was applied.

In contrast to the animals of the NaCl group of the present study, an additional rat with NaCl PCL implant treated with lower doses of toxins (triple injection of 62.5 ng TeT and administration of 7.4 mg CoCl_2_) survived without local application of VPA more than three weeks. Continuous video-ECoG monitoring in this rat documented frequent unprovoked clonic seizures for 12 days after surgery (Figure 7). 

In addition, in a sham-operated rat (without application of TeT/CoCl_2_ and PCL implant) no interictal epileptic activity and no subclinical or clinical seizures were detected by video-ECoG during three weeks after surgery. 

## 4. Discussion

In rats with TeT/CoCl_2_ application onto the primary motor neocortex, local VPA significantly enhanced survival in rats, possibly by focal suppression of epileptic activity or by counteracting epileptogenesis, as discussed in the following.

We here describe for the first time the elaborated methodology and the electrocorticographic and semiological characteristics of a new approach towards a neocortical epilepsy model in rats. This emerging model has been developed in an attempt to establish a valid tool for the evaluation of local therapeutic strategies in extrahippocampal epilepsies. It combines a triple injection of TeT and a circumscribed application of CoCl_2_ onto the right primary motor cortex. 

In contrast to other groups reporting on rat neocortical epilepsy models, we did not employ epidural, that is, extracortical, electrodes [3–7, 10–14] for intracranial EEG recordings or only a single, focal depth electrode [8], but a unique array of three intracortical depth electrodes which were placed both inside the TeT/CoCl_2_ application site and bilaterally outside of this focus. 

Driven by a clinical approach in the visual analysis of intracranial EEG and seizure semiology (similar to that applied for invasive video-EEG monitoring in humans), we were able to document frequent clear-cut interictal and ictal epileptic activity as well as recurrent spontaneous seizures in the major part of TeT/CoCl_2_-treated rats. We renounced additional computer-based EEG analyses (e.g., frequency spectrum analyses) because such calculations may be hampered by the risk of erroneously involving artifacts or mistaking physiological for epileptic activity. 

Although some interanimal variability in electrocorticographic and behavioural seizure characteristics was observed, initial seizure semiology in all rats typically consisted of focal rhythmic clonic or tonic motor phenomena. In contrast to Nilsen et al. [8], we did not observe the alleged long-lasting mild behavioural seizures with bilateral facial myoclonic jerking and arrest, although up to the 9-fold dosage of TeT was injected. 

Why were the epileptic discharges in our TeT/CoCl_2_ approach predominantly found at electrodes outside the TeT/CoCl_2_ application site? A prominent contralateral epileptic focus following unilateral frontal but not parietal cobalt application was also described by Dow et al. [6]. Noteworthy, these authors also reported contralateral ictal twitching of the forelimb, leading to the assumption of a seizure onset still ipsilateral to the cobalt-treated frontal cortex. The missing congruence between the site of the neocortical lesion and the topography of the recorded intracranial epileptic activity can partly be explained by a sampling problem inherent to the use of a limited number of electrodes. Alternatively, it may reflect a CoCl_2_-induced necrotic process adjacent to the “focal” electrode. The implantation of multiple “focal” and “extrafocal” (including contralateral) depth electrodes, as established in our TeT/CoCl_2_ setup, might therefore be crucial for a reliable detection of interictal and ictal neocortical epileptic activity. 

Despite a rather small number of compared animals (5 in each group), we have proven that the majority (75%) of TeT/CoCl_2_-treated rats surviving the first postoperative day developed epilepsy with recurrent spontaneous neocortical seizures. Since Tamargo et al. [4] reported a lower epilepsy rate of only 48.5% after neocortical application of CoCl_2_ alone, though at higher dosage (20 mg instead of 15 mg), the additional triple injection of TeT in our novel approach may have increased epileptogenesis. On the other hand, after insertion of cobalt wires in the left motor cortex, Chang et al. [3] observed focal and secondary generalized seizures in all nine reported rats. Possibly, continuous video-ECoG monitoring, as opposed to our prolonged intermittent recordings, facilitated the documentation of seizures in the study of Chang et al. [3]. 

Overall, the course of the focal neocortical epilepsy induced in the TeT/CoCl_2_ model described here resembles that seen earlier in cobalt-treated rats [3–7, 10–13]. Following a latency period of one to five days, a peak of epileptic activity about one week after cobalt treatment with a marked decline of overt seizures afterwards was commonly observed. This decay is a clear disadvantage of cobalt-induced neocortical epilepsy models, as long-term epileptological investigations are hampered. Unfortunately, the pathophysiological background of the decay is not well understood. In the case of the present study, a concluding statement on the long-term course of the epilepsy induced by the combined neocortical application of TeT and CoCl_2_ is not yet possible, as only rats treated with the antiepileptic drug VPA survived longer than one week. However, in an additional rat with neocortical administration of only 187.5 ng TeT and 7.4 mg CoCl_2_, but no local VPA treatment, distinct clinical seizures have occurred far beyond one week after surgery. This also argues against the assumption that the documented seizures might be directly provoked by the initially administered toxins. Instead, toxin-induced secondary epileptogenic processes leading to recurrent spontaneous seizures are more likely. As shown in a sham-operated rat without TeT/CoCl_2_, the surgical procedure (including anesthesia) alone does not induce interictal or ictal epileptic activity. Currently, it remains speculative whether a possible idiosyncratic or supra-additive epileptogenic pathomechanism is specifically induced by the combination of TeT and CoCl_2_. 

We are aware that further studies with lower and presumably less lethal dosages of TeT and CoCl_2_ and with continuous long-term video-ECoG monitoring are necessary. These studies must further validate our TeT/CoCl_2_ approach as suitable animal model and will determine whether additional TeT, in comparison with the CoCl_2_ component alone, provides decisive advantages with respect to the induction rate of focal neocortical epilepsy and its duration. By means of thorough histopathological analyses, we are also planning a systematic evaluation whether the degree of epilepsy in the evolving model correlates with the extent of morphological lesions induced by TeT plus CoCl_2_ or whether traumatic neocortical injury induced by surgery may play an additional role in epileptogenesis.

### 4.1. Effects of VPA

#### 4.1.1. Local Epilepsy Therapy

Since its introduction for the treatment of epilepsy in 1963, VPA has confirmed its particular antiepileptic efficacy in a broad spectrum of seizure disorders. It is a water soluble low molecular weight drug with several mechanisms of action (for pharmacological review, see [15]). For clinical use, various formulations for oral or systemic administration are available. Applying a novel 3D-bioplotting technology we have recently constructed VPA PCL implants with VPA release kinetics appropriate for the investigation of early effects in local epilepsy therapy [1]. The VPA PCL implants (10% w/w), which were also used in the present study, released already 77% of the maximum possible amount within the first 24 hours when superfused *in vitro*; 88% of VPA were released after 4 days [1]. *In vivo*, however, these high release rates may be lower since only one side of the implant contacts the brain surface. From a technical point of view, the implantation of VPA PCL devices in our new TeT/CoCl_2_ procedure can easily be integrated during surgery, thus ensuring a reliable local VPA release directly onto the neocortical epileptogenic focus. 

Preliminary experiments in our laboratory had indicated that locally applied VPA potentially suppressed interictal epileptic activity in the emerging TeT/CoCl_2_ rat model. The present randomized study, therefore, was aimed at examining the antiepileptic effects of local VPA in a current version of this TeT/CoCl_2_ model, using NaCl PCL-treated rats as controls. Animals of both the VPA and the NaCl group exhibited clear-cut interictal epileptiform potentials and neocortical seizures, as described above. However, statistical analyses of variables beyond the below-detailed survival rate were not possible as only few vehicle-treated rats were eligible for sufficient longitudinal video-ECoG recordings. 

A goal of further research will be, therefore, to establish a balanced TeT/CoCl_2_ dose regime that allows prolonged survival of epileptic rats and enables a statistically valid comparison of immediate and long-term AED effects on interictal and ictal epileptic activity. Further investigations should also compare local versus systemic application of VPA and other AEDs in a finally elaborated TeT/CoCl_2_ model. 

#### 4.1.2. Survival

In the present randomized study, local administration of VPA directly onto the epileptogenic focus clearly prevented early death which was obvious in control rats. Note that the Kaplan-Meier survival function from life-time data is much more meaningful than the percentage survival at a random time point. The differences in survival between the VPA and the NaCl group can neither simply be attributed to perioperative surgical complications nor to any differing surgical variables or postoperative conditions. 

The reasons for the enhanced survival due to focal application of VPA are yet unexplained. It might be suspected that a VPA-mediated prevention of lethal seizures played a role, as all witnessed deaths in our study occurred during status epilepticus or were seizure-related. 

While Brener at al. [16] reported a dose-dependent percentage of deaths up to 36% after neocortical TeT injections without specifying the cause, Nilsen et al. [8] did not observe any seizure-related deaths in their TeT model of neocortical epilepsy. Liang et al. [17] reported deaths following generalized seizures after neocortical TeT in 29% of rats. Death from status epilepticus has been documented after cortical application of 20 mg CoCl_2_ [4]. Overall, seizure-associated deaths in rats following neocortical epileptogenesis through TeT or CoCl_2_ are not rare. 

To our knowledge, reports on effects of VPA in rat TeT models of neocortical epilepsy are not available. However, there is some evidence that VPA might exert an antiepileptic effect in cobalt-treated animals. Van Duijn and Beckmann [18] described that subcutaneous or intravenous VPA did not suppress focal epileptic discharges produced by topical cobalt in the sensorimotor cortex of the cat but that intravenous VPA inhibited their spread from the epileptic focus to the contralateral hemisphere; corresponding VPA blood levels were between 0.3 and 0.5 *μ*g/mL. Walton and Treiman [19] reported that VPA given intraperitoneally in rats with neocortical cobalt lesions dose-dependently controlled status epilepticus provoked by homocysteine thiolactone; however, VPA did not completely prevent interictal epileptic activity even when serum levels exceeded 1000 *μ*g/mL. 

In a rat epilepsy model based on intraventricular injection of CoCl_2_ intraperitoneal VPA at high doses (300 mg/kg) neither antagonized seizures nor decreased mortality [20]. 

While the antiepileptic effect of VPA in cobalt-treated rats appears mild only after *systemic *application, it seems probable that the *local *VPA application in our experimental TeT/CoCl_2_ setup enhances the efficacy of this drug through targeted focal delivery. The concept of local therapy of neocortical epilepsy has already been studied earlier in rats using intracerebral phenytoin polymers adjacent to a CoCl_2_-induced epileptic focus [4]. Also the glutamate-receptor antagonist 1,2,3,4-tetrahydro-6-nitro-2,3-dioxo-benzo[f]quinoxaline-7-sulfonamide (NBQX) applied onto a TeT-induced focus was reported to reduce seizure activity [8]. Focal neocortical application of VPA has not been reported so far. The particular release kinetics of the VPA PCL implants [1] used in the present study support the assumption that during the first days high concentrations of VPA were achieved directly in the circumscribed brain area exposed to TeT and CoCl_2_. The resulting antiepileptic efficacy might have contributed to a lower incidence of fatal seizures and a prolonged survival in the VPA group. Thus, we speculate that the effect on survival reported in the present study might not be unique to valproate but potentially shared by other antiepileptic agents. 

However, further investigations are necessary in order to determine whether in addition a specific antiepileptogenic effect of VPA (for review, see [21]) might play a decisive role when applied locally in the early phase of the epileptogenic process. In contrast, oral administration of standard VPA doses does not prevent or postpone epileptogenesis after traumatic brain injury in humans [22].

## 5. Conclusion and Perspectives

The novel TeT/CoCl_2_ approach towards a reliable animal model of focal neocortical epilepsy is a promising tool for video-ECoG-based preclinical studies to develop effective and well tolerated local pharmacotherapeutic strategies in neocortical epilepsies. Here, local VPA significantly enhanced survival in rats, possibly due to its antiepileptic/antiepileptogenic properties. Nevertheless, the neocortical TeT/CoCl_2_ epilepsy model needs further elaboration. 

In order to evaluate the potential impact of local epilepsy therapy in humans, we are currently examining the effects of local administration of VPA onto circumscribed neocortical epileptic foci in patients scheduled for tailored epilepsy surgery after a presurgical work-up with subdural electrodes.

## Abbreviations

AED: Antiepileptic drug
CoCl_2_: Cobalt(II) chloride hexahydrate
ECoG: Electrocorticography
EEG: Electroencephalography
EMG: Electromyography
NaCl: Sodium chloride
PCL: Polycaprolactone
TeT: Tetanus toxin
VPA: Valproate.
